# The Tincture of the Chloride of Iron

**Published:** 1895-12

**Authors:** G. S. Martin

**Affiliations:** Toronto Junction, Ont.


ARTICLE VI.

### THE TINCTURE OF THE CHIORIDE OF IRON.

BY G. S. MARTIN, L. D. S., D. D. S., TORONTO JUNCTION, ONT.

Read before the Toronto Dental Society.

Were the subject of my paper tonight "Iron," I would perhaps be inclined to write an essay dealing with the therapeutics of the iron preparations, internally as a tonic and externally as a styptic and astringent. I might recall to your mind that iron constitutes a necessary integral part of the red blood corpuscle, and may thus be said to be a food rather than a medicine. I might speak of its use in the treatment of such conditions as anæmia, and neuralgia resulting from anæmia, or of its great value as an aid to the appetite and digestion. But finding myself confined to one of the many preparations of iron—one for which the dentist has little or no use in his office practice—I am convinced that your committee had not in view a dissertation, however learned, on iron and its value as a styptic tonic.

In what way are we as dentists so interested in the tincture of the chloride of iron as to spend an evening in a discussion of it? Not as a styptic, I am sure, as for this purpose we have in the solution of the subsulphate (known as Monsel's) a much more efficient remedy As a tonic I find on enquiry among physicians and druggists of my acquaintance, that the tincture of the chloride is the most used of all the iron remedies, it being one of the most efficient and active preparations, and having the additional cardinal virtue of being the cheapest. The works on therapeutics tell us that the objections to its use are its constipating effects and its corrosive action on the teeth. It is to this corrosive action that I wish specially to direct your attention to-night.

In order to a thorough understanding of the question let us ask, What is tincture of the chloride of iron? First, in brief, its source: Iron wire is heated with H. Cl. and the ferrous chloride thus formed ($Fe.Cl._2$) is converted by further heating with H.Cl. and $H.NO._3$ into ferric chloride ($Fe._2 Cl._6$). This ferric chloride diluted with three volumes of rectified spirits gives the tinctura ferri perchloride of the B. P. In physical properties it is a bright yellow or reddish liquid with a strong odor of ether, very strongly astringent and corrosive in its effects and having an acid reaction.

This is the preparation whose effects as witnessed at the dental chair we wish to discuss to-night. I doubt if there is any one who will deny that this preparation has this corrosive effect, but if there are any such a simple experiment will, I think, serve to convince them. A sound tooth placed for a few hours in a solution of one drachm of tincture to one ounce of water will be found on examination so affected that the enamel may be readily scraped away.

In a practice dating back only a comparatively short time I have many times already heard the words, "Oh dear, I had such good teeth until I began taking iron." I have no doubt those of you who have been in practice some years can recall instances where, after you have put a patient's teeth in a splendid condition, they return to you after an absence of a few months, and to your surprise exhibit the ravages of extensive decay. On enquiry you have found that the patient has been using a medicine containing tincture of iron. Some years ago Dr. Smith, of Edinburgh, Scotland, conducted a series of experiments from which he concluded that of all the salts of iron the most corrosive were the tincture of the chloride, and the sulphate. The authorities, so far as I could find, throw very little light on the nature of the chemical action that takes place any more than to say that it is undoubtedly due to the action of the free acid present. Attfield, in his "Pharmaceutical Chemistry," says that it is practically impossible to obtain a preparation of ferric chloride free from an excess of H. Cl. It is noticed on experimenting on teeth in the test tube that the first appearance of change is a white spot, which rapidly extends over the whole surface of the tooth. This would indicate disintegration due to a mineral acid.

Admitting, then, this disastrous effect on the tooth, we come to the question: What is our duty as dentists? This question is one needing to be handled with great delicacy, involving as it does that other much-vexed question, the relations existing between the physician and the dentist and it is with some timidity that I approach it. The conscientious dentist will not be content only to repair damage done, but will strive by every means in his power to prevent the occurrence or recurrence of any further damage. If he felt that the use of vulcanite or amalgam would seriously endanger the health of his patient by producing a species of blood poisoning, he would abandon the use of these articles, though they might seem indispensable to the practice of dentistry. The conscientious physician, judging from the same professional and humanitarian standpoint, will be careful that in treating one condition he does not seriously affect some other organ or part of the system of his patient.

All the works on materia medica and therapeutics that I have been able to consult in preparing this paper, very distinctly say that this preparation of iron is specially injurious to the teeth, and should be used with great caution, so that if physicians are guided by recognized standards of medical practice they will not neglect, when they prescribe it, to give the patient such directions as will enable him to intelligently guard against evil results. From enquiries made and from my own observations, I am led to believe that there is a lamentably large class of physicians who, judged by these standards, are not conscientious in their treatment of patients. Druggists with whom I have conversed inform me that a large percentage of "iron prescriptions" contain no caution whatever as to taking. During the past month I found two patients of mine taking an iron preparation prescribed, and in at least one case dispensed by a physician. No directions had been given and the patients did not know that any bad results might follow.

Even when physicians do their duty in this matter it is doubtful very often if the result is satisfactory. Let us look at some of the means employed. The medicine is prescribed in a very dilute form, in the hope that thus the injurious effects will be avoided. The experiments of Dr. Giblin, of Boston (to whose paper [International '92] before the Harvard Odontological I am deeply indebted), go to show that to dilute the tincture of iron with water is to increase its injurious effects. A tooth immersed in a strong solution will become incased in a hard coating of oxide, which will at once act as a protection from free acid, while the oxide formed on a tooth immersed in a weak solution is flocculent and non-protective. Even when patients are instructed by their physician to use a tube I question if many of them will exercise sufficient care to make it of much use, as the tube must be carried well back in the teeth to insure success.

That there is an increasing interest being taken in this important subject is evident from the number of proprietary preparations advertised in our drug journals, each claiming to have preserved all the promptness and efficiency of the tincture and at the same time to have eliminated all the dangerous effects. I fancy the price of these preparations will be an effectual barrier to their general use. The use of alkaline gargles before and after taking the medicine seems to commend itself to me as the best way of counteracting the bad effects. It certainly is not for us as dentists to say to physicians what remedies they shall use, as we cannot be expected to trace through the whole system the action of a particular drug: but when the results of their treatment so seriously mar our work, and if by care these results might be avoided, then we are surely at liberty to criticise this want of care and to tell the physicians of our patients how, in our opinion, these results might be avoided,—*Dominion Dental Journal.*

